# Increased Carotid Intima-Media Thickness and Reduced Distensibility in Human Class III Obesity: Independent and Differential Influences of Adiposity and Blood Pressure on the Vasculature

**DOI:** 10.1371/journal.pone.0053972

**Published:** 2013-01-16

**Authors:** Xiao L. Moore, Danielle Michell, Sabrina Lee, Michael R. Skilton, Rajesh Nair, John B. Dixon, Anthony M. Dart, Jaye Chin-Dusting

**Affiliations:** 1 Baker IDI Heart and Diabetes Institute, Melbourne, Victoria, Australia; 2 The Boden Institute of Obesity, Nutrition, Exercise & Eating Disorders, The University of Sydney, Camperdown, New South Wales, Australia; 3 Obesity Research Unit, Department of General Practise, Monash University, Clayton, Victoria, Australia; 4 Alfred Heart Centre, Alfred Hospital, Prahran, Victoria, Australia; Universidad Pablo de Olavide, Centro Andaluz de Biología del Desarrollo-CSIC, Spain

## Abstract

Carotid intima-media-thickness (cIMT) and carotid distensibility (distensibility), structural and functional properties of carotid arteries respectively, are early markers, as well as strong predictors of cardiovascular disease (CVD). The characteristic of these two parameters in individuals with BMI>40.0 kg/m^2^ (Class III obesity), however, are largely unknown. The present study was designed to document cIMT and distensibility in this population and to relate these to other factors with established association with CVD in obesity. The study included 96 subjects (65 with BMI>40.0 kg/m^2^ and 31, age- and gender-matched, with BMI of 18.5 to 30.0 kg/m^2^). cIMT and distensibility were measured by non-invasive high resolution ultrasonography, circulatory CD133^+^/KDR^+^ angiogenic cells and endothelial microparticles (EMP) by flow cytometry, and plasma levels of adipokines, growth factors and cytokines by Luminex immunoassay kits. The study results demonstrated increased cIMT (0.62±0.11 mm *vs.* 0.54±0.08 mm, *P = 0.0002*) and reduced distensibility (22.52±10.79 10^−3^kpa^−1^ *vs.* 29.91±12.37 10^−3^kpa^−1^, *P<0.05*) in individuals with BMI>40.0 kg/m^2^. Both cIMT and distensibility were significantly associated with traditional CVD risk factors, adiposity/adipokines and inflammatory markers but had no association with circulating angiogenic cells. We also demonstrated, for the first time, elevated plasma EMP levels in individuals with BMI>40.0 kg/m^2^. In conclusion, cIMT is increased and distensibility reduced in Class III obesity with the changes predominantly related to conventional CVD risk factors present in this condition, demonstrating that both cIMT and distensibility remain as CVD markers in Class III obesity.

## Introduction

Carotid intima-media-thickness (cIMT) and carotid distensibility (distensibility) represent structural and functional properties of carotid arteries respectively. Both increased cIMT, a noninvasive measure of subclinical atherosclerosis, and reduced distensibility, an indicator of regional artery stiffness, are independent predictors of future cardiovascular events [1], [2]. Importantly, a combined assessment of the two allows for a better analysis of the individual atherosclerotic burden and improved prediction of aortic atherosclerosis [3].

Increased cIMT or decreased distensibility has been linked to hypertension [4], diabetes mellitus [5] and obesity [6]–[10], determinant risk factors for cardiovascular disease (CVD) [11]–[14]. The occurrence of these three co-morbidities is linked with chronic low-grade inflammation. Furthermore insulin resistance present in obesity is believed to be a principal contributor to this link. The inter-relation between adipogenesis, inflammation, insulin resistance, hypertension and diabetes mellitus remains a current focus of obesity research. Nevertheless higher CVD incidences are evident in hypertensive and/or diabetic obese compared to non-obese counterparts. The prevalence of obesity is rising at an alarming rate worldwide. Moreover the prevalence of Class III obesity, defined as BMI≥40.0 kg/m^2^, is increasing at an even steeper rate [15], [16]. cIMT and distensibility in Class III obesity, however, are largely undocumented with only two papers providing both cIMT and distensibility data in people with BMI≥40.0 kg/m^2^ [17], [18]. Similarly, little information is available in Class III obesity on novel biomarkers of CVD, such as circulatory angiogenic cells [19] or endothelial microparticles [20].

The main objective of this study was therefore to document cIMT and distensibility in Class III obese subjects compared with a non-obese cohort, and to examine and compare traditional CVD risk factors (CVRF) and novel CVD biomarkers between the two populations. We hypothesized that cIMT and distensibility remain useful as CVD markers in the severely obese population despite technical difficulties that may be encountered and verified this by determining the association of cIMT and distensibility with other established CVRF in Class III obesity.

## Materials and Methods

### Ethics Statement

The study protocol was approved by the institutional ethics committee of Alfred Healthcare (#158/06), and informed written consent was obtained from each participant.

### Study Population and Design

A total of 96 subjects (31 non-obese controls: BMI 18.5 to 30.0 kg/m^2^ and 65 class III obesity: BMI>40.0 kg/m^2^) were included in the study. Class III obesity subjects were recruited via the Obesity Research Groups at Monash University while the age- and gender-matched non obese were from the Baker IDI BioBank database. Exclusion criteria were known coronary artery disease, cardiac failure, vascular brain disease, peripheral obstructive artery disease, significant renal or hepatic dysfunction and pregnancy. Subjects with current or past history of multiple myeloma, blood dyscrasia or any form of leukemia or lymphoma were also excluded.

All individuals underwent a physical examination and had their medical histories recorded. In brief, participants were measured for height, weight, waist and hip circumferences and blood pressure. 30 ml of peripheral blood was drawn for routine blood tests following a 12 hr fast and also analyzed for levels of plasma adipokines, growth factors and cytokines, circulating angiogenic cells (CD133^+^/KDR^+^ PBMCs & Hill-CFU) and endothelial microparticles (EMP). Routine blood tests were performed by the Alfred Pathology Department including a full blood count, hsCRP, glucose and a lipid profile (HDL, LDL, total cholesterol and triglycerides). cIMT and distensibility were examined using non-invasive high resolution ultrasonography.

The measurement of cIMT and distensibility were compared between the two groups. The associations of cIMT or distensibility with traditional CVRF (age, gender, BP, glucose and lipids etc) and adiposity/adipokines (BMI, waist:hip, adiponectin and leptin) were examined, as were their respective associations with inflammatory markers and circulating angiogenic cells. Plasma levels of EMP were measured in randomly selected subpopulations including both males and females (Class III obese = 15 and non-obese = 16) to determine vascular inflammation and integrity.

### Carotid Imaging and Measurement

The left and right common carotid arteries proximal to the carotid bifurcation were imaged through non-invasive high resolution ultrasonography using a Philips iE33 ultrasound system (Philips, Bothell, WA, USA) with a 11–3 MHz linear array transducer while the subject was at rest in a supine position. Briefly, the carotid arteries were imaged in longitudinal sections, 0–2 cm proximal to the carotid artery bifurcation, focusing on the far wall of the vessel. Two 10-second loops were captured for each of the left and right arteries and stored for offline analysis. cIMT was defined as the distance between the intima-lumen interface and the media-adventitia interface, and measured at end diastole (as determined from the simultaneous electrocardiogram recordings) over a 10 mm long portion of the vessel wall between 0–1 cm proximal to the carotid bulb. Diastolic and systolic diameters, for distensibility calculation, were determined as the smallest and largest diameter values during a cardiac cycle. An average of three measurements from consecutive cardiac cycles from each of the left and the right carotid artery was made, and the average of the left and right arteries was used for the final analysis. All measurements were conducted independently using an automatic edge detection system (Philips QLAB version 7.0) by two observers blinded to all participant information.

Carotid distensibility was calculated as (2Δd/ds)/ΔP in 10^−3^•kPa^−1^, where Δd is carotid internal diameter change between systole and diastole, ds is carotid systolic diameter and ΔP is pulse pressure [21].

### Determination of Plasma Adipokines and Cytokines

Plasma samples were collected and stored at −80°C until use. Plasma levels of adiponectin, leptin, IL-10 and SDF-1 were measured using Luminex immunoassay kits (Millipore, USA) as per manufacturer’s instruction. Briefly, the appropriate adipokines or cytokine standards, plasma samples (25 µL), and fluorescent conjugated, antibody-immobilized beads were added to wells of a pre-wet filtered plate and then incubated in dark overnight at 4°C. The following day, the plate was washed twice with wash buffer and then incubated with secondary detection antibody for 1 hr, followed by subsequent incubation with streptavidin-PE for 30 min. After the plate was washed twice again with wash buffer, it was run on the Luminex system (BioRad) with the addition of sheath fluid. Concentrations of different analytes in the plasma samples were determined by using respective standard curves generated in the assays.

### Measurement of Circulating Angiogenic Cells

#### Enumeration of peripheral blood CD133^+^/KDR^+^ PBMCs by flow cytometry

**Figure 1:**
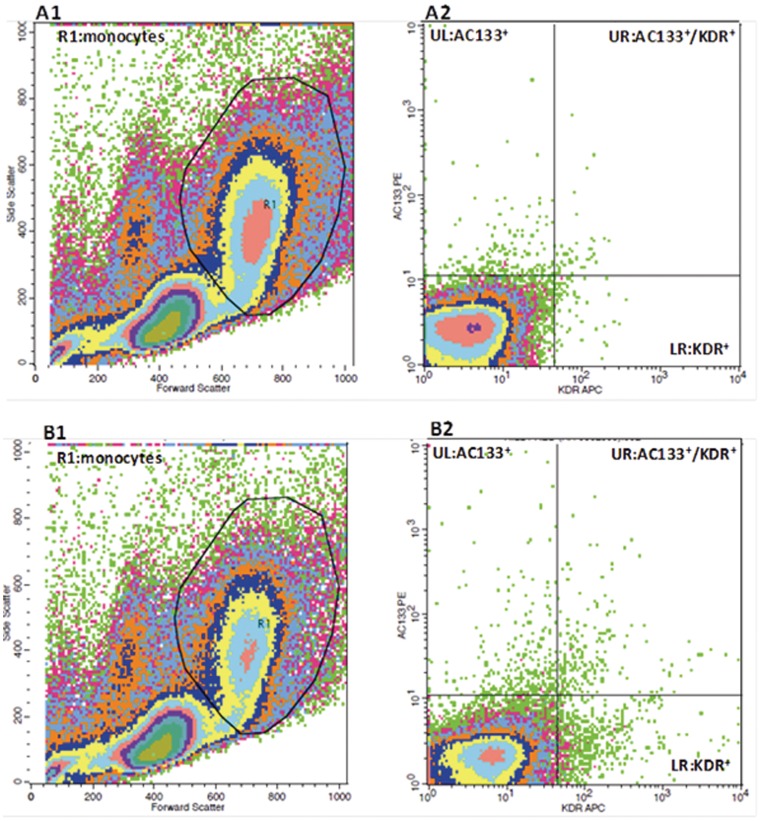
Illustration of FACS gating analysis of angiogenic cells (AC133^+^/KDR^+^PBMCs). Representative flow cytometric density plots demonstrating the gating protocol used to identify angiogenic cells: **A)** PBMCs stained with PE-conjugated and APC-conjugated mouse IgG (isotype controls) for non-specific fluorescent signals; **B)** PBMCs stained with antibodies against human CD133 (AC133) (PE-conjugated) and VEGFR-2 (KDR) (APC-conjugated) for AC133^+^/KDR^+^PBMCs; **A1, B1)** FSC-SSC density dot plots of Ficoll-isolated PBMCs and R1 was gated for monocytes; **A2, B2)** FL2 (PE)-FL4 (APC) density dot plots of R1-gated monocytes. Angiogenic cells = cells in B2 upper-right quadrant – cells in A2 upper-right quadrant.

Peripheral blood mononuclear cells (PBMCs), isolated from fresh venous blood by ficoll-gradient centrifugation, were used to quantitate the number of CD133^+^/KDR^+^ PBMCs by flow cytometry. In brief, 100 µl of PBMCs (1.5×10^7^/ml) was incubated with Fc-γ receptor blocking agent followed by 30 min incubation on ice with antibodies against human CD133 (PE-conjugated, Miltenyi Biotec, Germany) and VEGFR-2 (KDR) (APC-conjugated, R&D Systems, USA). PE- and APC-conjugated mouse IgG from the same manufacturers served as isotype controls. Following incubation, cells were washed with PBS and then fixed in 1% paraformaldehyde. Flow cytometry acquisition was performed on BD FACSCalibur™ using appropriate settings excluding debris and platelets as shown in Figure 1-A1 & Figure 1-B1. 10^6^ events per sample were collected within the R1 monocyte gates. Cells positive for both CD133 and KDR (upper right quadrants of the FL2-FL4 plots as shown in Figure 1-A2 & Figure 1-B2) were characterized as angiogenic cells. Results were expressed as percentage of CD133^+^KDR^+^PBMCs/PBMCs. Analysis was carried out by a blinded randomized approach in regard to patient profiles using the FlowJo software (Tree Star Inc, USA).

#### Hill-CFU assay

The ability to clonally expand and generate colonies in an endothelial-specific medium is considered a key functional feature of angiogenic cells. The Hill-CFU assay was performed using the commercially available kit, EndoCult^TM^ liquid medium Kit (Stem cells Technologies, USA) and used as per the manufacturer’s instructions. In brief, 5×10^6^ ficoll-isolated PMNCs were resuspended with 2 ml EndoCult medium and plated in a well of fibronectin-coated 6-well plates (BD Biosciences, USA), which were incubated for two days at 37°C, 5% CO_2_ with ≥95% humidity. After two days the non-adherent cells were harvested, counted and plated as at a density of 1×10^6^ cells per well onto a 24-well fibronectin-coated plate, which was then incubated at 37°C for another 3 days. Hill-CFUs, characterized by a central cellular cluster surrounded by emerging spindle-shaped cells were counted at day 5 in 24-well plates in a minimum of 3 wells per subject and the average count was recorded. Results were expressed as number of colony per well.

### Isolation and Identification of Circulating EMPs

EMPs are defined as CD31^+^/CD41^−^ particles sized between 0.1–1 µm in platelet-depleted plasma. They were determined by the analysis for the expression of surface antigens by flow cytometry. In brief, 500 µl of completely thawed plasma was centrifuged at 16000 g for 5 min at 4°C to deplete platelets or any cell debris. The top 450 µl of plasma was transferred into a fresh tube, which was centrifuged again at 16000 g for 30 min at 4°C. The top 250 µl plasma was carefully removed and the remaining 200 µl vortexed and used for FACS analysis. Following a 15 min incubation with 50 µl Fc-γ receptor blocking agent (Miltenyi Biotec, Germany) at room temperature to reduce non-specific binding, half of the treated plasma was incubated with antibodies against human CD31 (Alexa647-conjugated, BD Biosciences, USA) and CD41 (PE-conjugated, BD Biosciences, USA). The other half was incubated with Alexa647- and PE-conjugated mouse IgG from the same manufacturer served as isotype controls. At the end of 20 min incubation, 300 µl of double filtered 1% Formaldehyde/0.2% FBS/PBS (filtered through a 0.2 and then a 0.1 µm membrane filter before use) was added for fixation and 50 µl of diluted calibration beads (BD Biosciences, USA) was added for EMP calculation and size reference. Each sample and its corresponding control were counted on BD FACSCalibur™ (BD Biosciences, USA) for 5 min.

**Figure 2:**
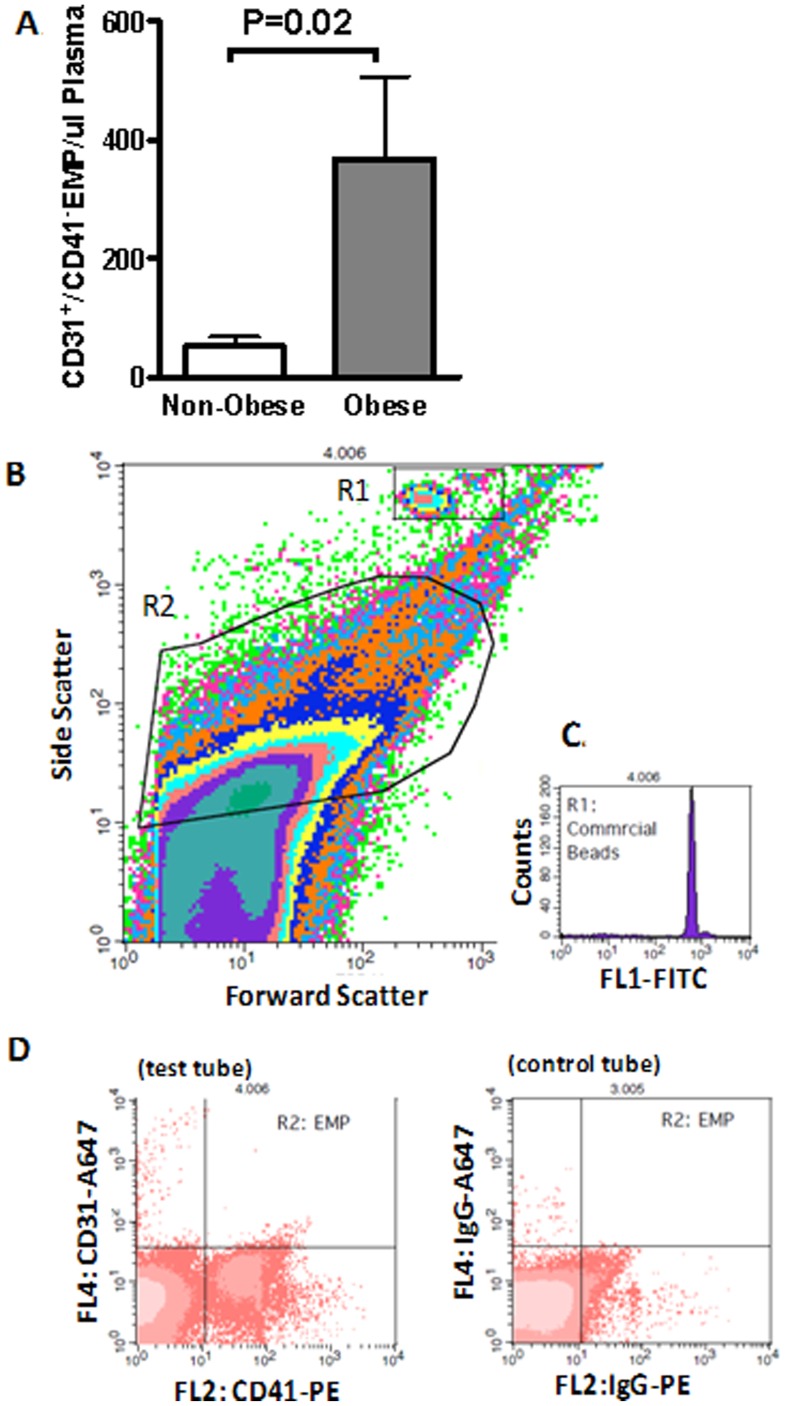
Elevated levels of circulating CD31^+^/CD41^−^ EMP in obesity. **A)**Quantification of circulating EMP levels in plasma. Data presented as mean±SEM. **B)** Representative flow cytometric density plots demonstrating the gating protocol used to identify EMP (R2) and bead populations (R1); **C)** Representative flow cytometric histogram of commercial beads; **D)** Representative flow cytometric dot plots demonstrating EMP population with a negative staining of CD41-PE but positive staining of CD31-Alexa 647 and its corresponding staining of isotype-controls.

For flow cytometry counting, EMP gate (R2 as shown in Figure 2-B) was pre-defined using commercial beads sized at 0.1 and 1 µm (Sigma-Aldrich, USA). Only events included within this gate were further analysed for fluorescence signal as shown in Figure 2-D. For EMP enumeration, a formula was used based on the concentration of the added calibration beads [22], which discriminated themselves from the EMP population on the FSC-SSC cytogram (R1 as shown in Figure 2-B and Figure 2-C). All counting data were then processed with a blinded randomized approach using BD CellQuest Pro (BD Biosciences, USA). Results are presented as number of CD31^+^/CD41^−^ EMP per µl of plasma.

### Statistical Analysis

Logarithmic transformations were applied if appropriate to skewed data following histogram analyses and Kolmogorov-Smirnov test. Transformed data are expressed as geometric mean (95% CI) and non-transformed data are expressed as mean ± SD. Comparisons between the non-obese and Class III obese groups were performed by two tailed Student’s t-test. Bivariate correlation analysis of adiposity (BMI and waist:hip), carotid variables (cIMT and distensibility) and blood pressure (SBP) was performed to define each crude association with other variables measured. Multivariable linear regression models were then constructed with the use of important covariates concluded from correlation analysis (*P<0.1*), in a hierarchal fashion, to elucidate independent determinants of cIMT and distensibility. Model 1 was adjusted for traditional CVRF (age, SBP, BP-med, fasting glucose and triglycerides), while Model 2 for traditional CVRF and adiposity/adipokines (BMI, adiponectin and leptin), and Model 3 for traditional CVRF, adiposity/adipokines and inflammatory markers (hsCRP, IL10 and WBC). Multivariable analysis was also repeated with no BP adjustment to further assess reliability of independent association between SBP and distensibility, since distensibility is a derivative parameter related to pulse pressure. For the same purpose, multivariable analysis of distensibility was again carried out separately in non-hypertensive and hypertensive subjects. In addition, multivariable analysis of SBP was performed as well. All statistical analyses were performed with SPSS version 12.0 for Windows and a probability value *P<0.05* was considered statistically significant.

## Results

**Figure 3:**
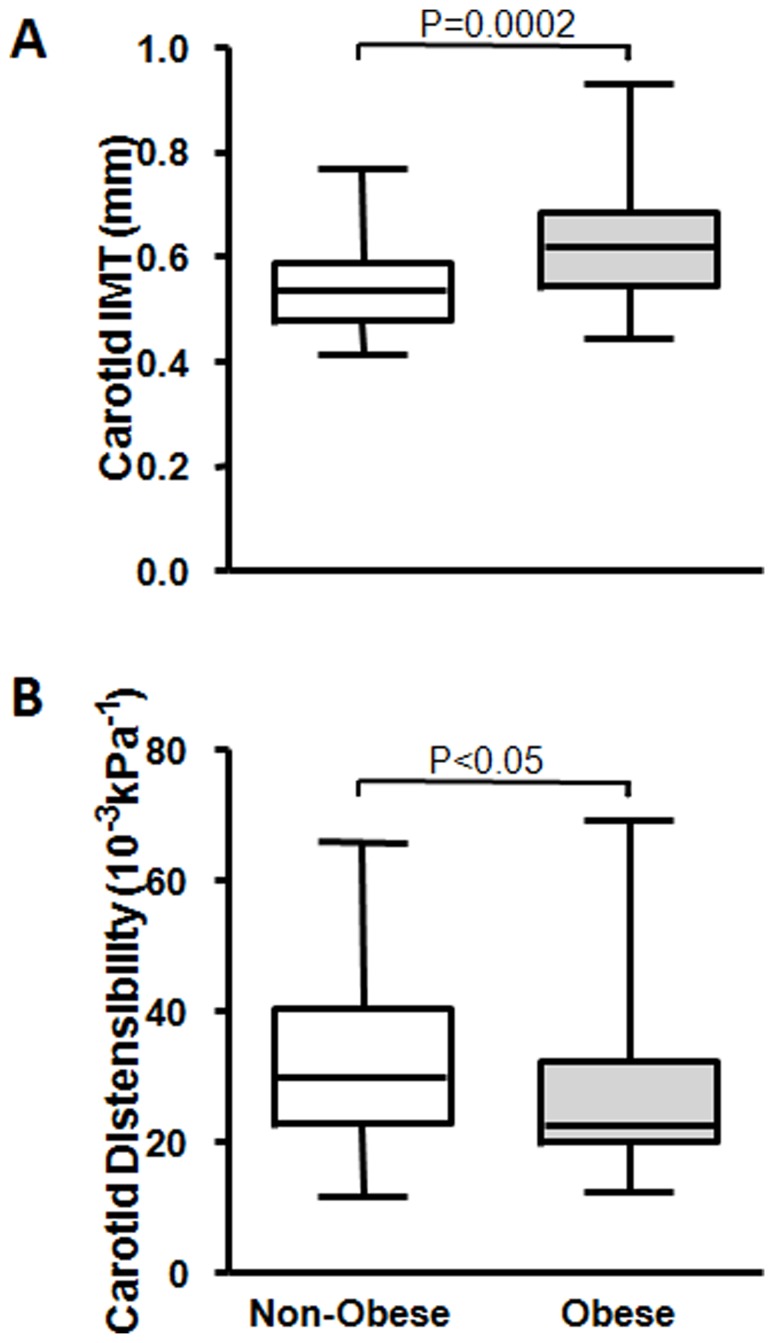
Increased cIMT (A) and reduced distensibility (B) in obesity. Median, minimum and maximum values of cIMT and distensibility.

The demographic, anthropometric, clinic and laboratory characteristics of the 96 subjects included in the study are shown in Table 1. Except for age, gender and smoking history, the 31 non-obese and 65 Class III obese subjects presented as two distinctive phenotypes in respective to all parameters related to traditional CVRF, adiposity, and plasma levels of circulating adipokines as well as inflammatory markers. All traditional CVRF measured were significantly worse in the Class III obese group. The heavier atherosclerotic burden in the Class III obese subjects was demonstrated both through increased cIMT (*P = 0.0002*) and reduced distensibility (*P<0.05*) in comparison to their age- and gender-matched non-obese counterparts (Figure 3-A & Figure 3-B). Furthermore, significantly higher levels of circulating EMP (*P = 0.02*) indicated vascular inflammation and a compromised integrity of vascular endothelium in Class III obese subjects (Figure 2-A).

**Table 1:** Anthropometric, clinical and biochemical characteristics.

	Non-obese	Severely Obese	*P*
	(n = 31)	(n = 65)	
Age, y	48.13±11.16	46.44±8.73	0.42
Male sex, %	48	52	0.72
Smoking, %	52	52	0.97
Systolic blood pressure, mmHg	120.47±16.78	139.9±17.59	<0.0001
Diastolic blood pressure, mmHg	73.75±10.98	85.88±11.22	<0.0001
Mean arterial pressure, mmHg	88.43±11.98	103.06±11.82	<0.0001
Pulse pressure, mmHg	46.72 (42.6, 50.8)	54.03 (50.4, 57.6)	0.014
Hypertension, %	26	60	0.001
Blood Pressure-Medication, %	13	50	<0.0001
Fasting glucose, mmol/L	4.82 (4.7, 5.0)	6.33 (5.7, 7.0)	<0.0001
Type 2 Diabetes, %	7	56	<0.0001
Total cholesterol, mmol/L	5.28 (4.9, 5.7)	5.18 (5.7, 7.0)	0.70
HDL cholesterol, mmol/L	1.56 (1.4, 1.8)	1.17 (1.1, 1.2)	<0.0001
LDL cholesterol, mmol/L	3.25±0.87	3.17±0.99	0.69
Triglycerides, mmol/L	1.02 (0.9, 1.2)	1.88 (1.7, 2.1)	<0.0001
Body mass index (BMI), kg/m^2^	25.51±2.61	45.46±5.47	<0.0001
Waist:Hip	0.85±0.09	0.97±0.09	<0.0001
Leptin, ng/ml	11.46 (7.2, 15.7)	41.0 (33.8, 48.1)	<0.0001
Adiponectin, µg/ml	48.90 (39.4, 58.4)	14.6 (9.8, 19.5)	<0.0001
White blood cells (WBC), 10^9^/L	5.47±1.19	7.58±1.83	<0.0001
hsCRP, mg/L	1.19 (0.8, 1.5)	6.41 (5.6, 7.2)	<0.0001
Interleukin-10 (IL-10), pg/ml	30.45 (13.7, 47.2)	6.7 (4.1, 9.3)	0.007
CD133^+^KDR^+^ PBMC, %	0.34 (0.32, 0.35)	0.34 (0.32, 0.36)	0.91
Hill-colony forming units	4.06 (1.7, 6.4)	11.7 (7.0, 16.4)	0.005
SDF-1, pg/ml	1492.31±554.09	2929.83±2387.46	<0.0001

Data are expressed as means±SD (non-transformed data) or geometric means (95% CI) (transformed data). HDL indicates high-density lipoprotein; LDL, low-density lipoprotein; hsCRP, high-sensitivity C-reactive protein; PBMC, peripheral blood mononuclear cells; SDF-1, stromal cell-derived factor-1.

As expected, significantly elevated plasma leptin and suppressed adiponectin, a typical adipokine phenotype of obesity, were shown in Class III obese subjects (Table 1), as was the inflammatory profile of much higher levels of plasma hsCRP but lower IL10. Both measures of adiposity, BMI and waist:hip, closely correlated to status of metabolic syndromes (blood pressure, glucose, type 2 diabetes, HDL and triglycerides) and inflammation (Table 2).

**Table 2:** Bivariate correlation between BMI, Waist:Hip, SBP, cIMT and CD with other covariates.

	BMI		Waist/Hip		SBP		cIMT		CD	
	r	p-value	r	p-value	r	p-value	r	p-value	R	p-value
Age	−0.080	0.48	−0.056	0.60	0.084	0.43	**0.291**	**0.005**	−**0.323**	**0.002**
Gender	0.123	0.28	**0.654**	**<0.0001**	0.143	0.17	0.065	0.54	−0.115	0.29
Smoking	0.062	0.59	0.193	0.09	0.114	0.32	0.128	0.27	−0.127	0.28
SBP	**0.587**	**<0.0001**	**0.409**	**<0.0001**	–	–	**0.323**	**0.002**	−**0.643**	**<0.0001**
Hypertension	**0.397**	**<0.0001**	**0.332**	**0.001**	**0.593**	**<0.0001**	**0.246**	**0.020**	−**0.383**	**<0.0001**
BP-Med	**0.359**	**0.001**	**0.406**	**<0.0001**	**0.254**	**0.024**	0.213	0.06	−**0.256**	**0.029**
Type 2 Diabetes	**0.434**	**<0.0001**	**0.372**	**0.001**	**0.385**	**<0.0001**	**0.307**	**0.005**	−**0.305**	**0.007**
Fasting glucose	**0.338**	**0.003**	**0.314**	**0.003**	**0.236**	**0.025**	**0.343**	**0.001**	−**0.267**	**0.015**
HDL cholesterol	−**0.472**	**<0.0001**	−**0.540**	**<0.0001**	−0.158	0.13	−0.143	0.18	0.026	0.81
LDL cholesterol	0.014	0.91	0.140	0.19	0.144	0.17	0.107	0.32	0.019	0.86
Triglycerides	**0.533**	**<0.0001**	**0.448**	**<0.0001**	**0.278**	**0.014**	**0.256**	**0.026**	−0.221	0.06
BMI	–	–	**0.633**	**<0.0001**	**0.580**	**<0.0001**	**0.422**	**<0.0001**	−**0.379**	**0.001**
Waist:Hip	**0.633**	**<0.0001**	–	–	**0.409**	**<0.0001**	**0.385**	**<0.0001**	−**0.289**	**0.008**
Adiponectin	**−0.601**	**<0.0001**	**−0.460**	**<0.0001**	**−0.403**	**<0.0001**	**−**0.181	0.12	**0.301**	**0.010**
Leptin	**0.733**	**<0.0001**	**0.301**	**0.008**	**0.383**	**0.001**	0.220	0.056	**−0.282**	**0.015**
hsCRP	**0.808**	**<0.0001**	**0.530**	**<0.0001**	**0.432**	**<0.0001**	**0.335**	**0.003**	**−0.337**	**0.004**
IL-10	**−0.467**	**<0.0001**	**−0.316**	**0.005**	**−0.265**	**0.019**	−0.168	0.15	0.079	0.51
WBC	**0.550**	**<0.0001**	**0.429**	**<0.0001**	0.216	0.051	0.170	0.13	−0.115	0.32
CD133^+^KDR^+^PBMC,%	−0.003	0.98	0.026	0.81	0.120	0.25	0.128	0.23	0.19	0.87
Hill-colony forming units	0.141	0.28	0.145	0.22	0.116	0.33	−0.116	0.33	0.023	0.85
SDF-1	**0.309**	**0.006**	0.110	0.34	0.198	0.08	0.126	0.28	−0.096	0.42

BP-Med indicates blood pressure related medication, SBP, systolic blood pressure.

On the other hand, there was no difference in the number of circulatory angiogenic cells (CD133^+^KDR^+^PBMC) between obese and non-obese populations, despite significantly higher colony-forming capacities (Hill-CFU) in Class III obese subjects (Table 1). Plasma levels of SDF-1, a critical cytokine mobilizing angiogenic cells, were nearly doubled (*P = 0.003*) in subjects with BMI>40 kg/m^2^.

cIMT significantly correlated with age, BMI, waist:hip ratio, hsCRP, and status of the metabolic syndrome (Table 2). These correlations were also true for distensibility. As well, distensibility was also linked to BP-medication and plasma levels of leptin and adiponectin.

Subjects with hypertension were defined, in this study, for those who either presented with elevated blood pressure (≥140/90 mmHg) at the time of BP measurement or were taking antihypertensive medications (BP-med) or had a history of hypertension. Blood pressure was significantly elevated in Class III obese subjects, and multivariate regression analysis revealed that SBP was independently associated with BMI (β = 0.879, *P<0.0001*) and plasma levels of adiponectin (β = −0.304, *P = 0.049*) in this cohort. Multivariate regression analysis of cIMT and distensibility, therefore, was performed with and without BP adjustment (Table 3). The results showed that age and BMI were independently associated with cIMT regardless of BP adjustment (Table 3). In contrast, carotid distensibility was independently associated with age, BP-medication and plasma levels of adiponectin when adjusted for SBP, which was also associated with distensibility, but only with age and plasma adiponectin when not adjusted for BP. In further regression analysis for distensibility stratified by hypertensive status, age (non-hypertensive: β = −0.562, *P<0.0001*; hypertensive: β = −0.465, *P = 0.007*) and plasma adiponectin (non-hypertensive: β = 0.527, *P = 0.001*; hypertensive: β = 0.518, *P = 0.023*) were independently associated with distensibility in non-hypertensive and hypertensive groups, in addition to SBP in the hypertensive group (β = −0.465, *P = 0.007*).

**Table 3:** Multivariable linear Regression Analyses.

		cIMT		CD	
Model		β	p-value	β	p-value
**SBP adjusted-Model 1**					
Age	**0.271**		**0.017**	−**0.351**	**<0.0001**
SBP	0.199		0.08	−**0.585**	**<0.0001**
**Model 2**					
Age	**0.313**		**0.009**	−**0.418**	**<0.0001**
SBP	0.031		0.81	−**0.619**	**<0.0001**
BP-med	0.065		0.56	−**0.202**	**0.020**
BMI	**0.520**		**0.020**	−**0.372**	**0.026**
Adiponectin	0.004		0.98	**0.308**	**0.010**
**Model 3**					
Age	**0.359**		**0.006**	−**0.421**	**<0.0001**
SBP	−0.015		0.91	−**0.592**	**<0.0001**
BP-Med	0.042		0.72	−**0.215**	**0.018**
BMI	**0.723**		**0.013**	−0.300	0.16
Adiponectin	−0.076		0.66	**0.378**	**0.004**
**No BP adjustment-Model 1**					
Age	**0.288**		**0.013**	−**0.424**	**<0.0001**
**Model 2**					
Age	**0.329**		**0.005**	−**0.536**	**<0.0001**
BMI	**0.329**		**0.004**	−0.096	0.58
Adiponectin	0.000		0.999	**0.400**	**0.008**
**Model 3**					
Age	**0.364**		**0.004**	−**0.568**	**<0.0001**
BMI	**0.661**		**0.003**	−0.322	0.12
Adiponectin	−0.061		0.72	**0.552**	**0.001**

Model 1 was adjusted for traditional CVRF (age, SBP, BP-med, fasting glucose and triglycerides), while Model 2 for traditional CVRF and adiposity/adipokines (BMI, adiponectin and leptin), and Model 3 for traditional CVRF, adiposity/adipokines and inflammatory markers (hsCRP, IL10 and WBC). Results are expressed as standardized β and only factors showing significant association are listed.

## Discussion

The present study demonstrates that increased cIMT and reduced distensibility is observed in Class III obese subjects with no overt CVD conditions when compared to their age- and gender-matched non-obese counterparts. Changes in both cIMT and distensibility corresponded well with elevated traditional CVRF in this cohort. We also show that cIMT and distensibility are significantly associated with adiposity, adipokines and inflammatory markers, however, none had any connection with circulatory angiogenic cells.

Since obesity, and in particular abdominal obesity, is a major risk factor for CVD [23], tools to screen, monitor and predict CVD in this population can be very useful clinically. cIMT and distensibility, structural and functional parameters of carotid arteries, are early markers as well as strong predictors of CVD [1], [2]. While previous studies have demonstrated a strong association of increased cIMT and/or reduced distensibility with obesity [6]–[9], documentation of these two parameters of carotid artery in individuals with BMI>40 kg/m^2^ is lacking. Our finding that cIMT is increased and distensibility reduced in the Class III obese is consistent with the only other published studies that documented increased cIMT in 64 subjects with BMI of 42.3±4.3 kg/m^2^ [17], [18] and increased cIMT and decreased distensibility in 13 obese subjects with average BMI of 40.5±7 kg/m^2^ [18]. In addition, we show that cIMT is positively associated with age, adiposity, blood pressure, type-2 diabetes, hyperglycemia, dyslipidemia and hsCRP, while distensibility demonstrates negative associations with these covariates. These demonstrated associations verify that cIMT and distensiblity remain as CVD markers in Class III obesity.

Obesity is highly associated with the metabolic syndrome (MS), which is closely linked to cardiovascular morbidity and mortality [24]. Two of the most widely accepted MS criteria have been respectively promulgated by the World Health Organization (WHO) and the National Cholesterol Education Program (NCEP-ATP III). The principal distinction between the two is that that the NCEP-ATP III emphasizes CVD risks whereas the WHO focus on insulin resistance [25]. We used NCEP-ATP III criteria for this study. Based on the MS criteria defined by the NCEP-ATP III [25], about 86% of our Class III obese subjects had MS: dyslipidemia, hyperglycemia (diabetes), hypertension, or central obesity. This extremely high prevalence of MS was highly associated with BMI in our cohort as evidenced by the close association of BMI with various measures of MS status (Table 2). It is thus not surprising that BMI and/or plasma adiponectin was found to be independently linked to cIMT and distensibility, besides age which is a strong predictor of arterial remodeling [26], [27].

Hypertension and diabetes mellitus are common in obesity, which is also evident in this study. 60% and 56% of our Class III obese subjects were, respectively, hypertensive or diabetic in comparison to 20% and 7% in the non-obese group. Indeed in our obese subjects only 14% were free of both diabetes and hypertension. As both hypertension and diabetes are important in development of CVD, it is therefore difficult to apportion relative contributions to hypertension, diabetes or obesity per se. Further, artery stiffness and blood pressure are two closely related factors and distensibility is a derivative parameter of pulse pressure. Indeed, distensibility was revealed to independently associate with SBP and BP-medication besides age and plasma adiponectin when BP was adjusted in its multivariate regression analysis. The fact that an independent link between distensibility and plasma adiponectin stands regardless of the adjustment of BP demonstrates the essential role of adiposity in development of CVD suggesting the paramount importance of weight control in prevention and reduction of CVD. Obesity is also known to be characterized by a chronic systemic inflammatory state [28]. This was reflected by elevated plasma levels of CRP in our study. Plasma levels of CRP were strongly correlated with all important measures, cIMT, distensibility, BMI/waist:hip and as well as SBP, demonstrating a crucial role of inflammation in the development of obesity and obesity associated CVD. To further directly assess vascular inflammation and integrity, plasma endothelial microparticles (EMP) were examined and compared in randomly selected subpopulations including both genders from both groups. EMP are small membrane vesicles, 0.1–1 µm in diameter, which are released from the endothelium following endothelial cell activation or injury by a process of exocytotic budding of the plasma membrane [29] sometimes referred to as endothelial blebbing. Increased plasma EMP levels can be detected by FACS [30] and have been reported in various CVD conditions [29], [31]. In patients presenting a characterized endothelial dysfunction, levels of circulating EMP are inversely correlated with the amplitude of flow-mediated dilatation, independent of age and pressure [31], [32]. EMP has thus been suggested as a novel surrogate marker of endothelial injury, which precedes CVD, and hence a novel potential biomarker of CVD [20]. To our knowledge, this is the first report measuring EMPs in the Class III obese population and we show, for the first time, that circulating levels of EMP are significantly elevated in obesity.

In addition, we also examined plasma levels of angiogenic cells, an established cellular biomarker of CVD, and their associated cytokine SDF-1, a potent stimulator to mobilize angiogenic cells [33]. Decreased circulating levels of endothelial progenitor cells (EPC) have been used as an indicator of higher CVD risk [19]. We measured angiogenic cells in our study as CD133^+^/KDR^+^ peripheral blood mononuclear cells (PBMCs) by FACS. In comparison to EPC, commonly defined as CD34^+^/KDR^+^ PBMCs, CD133^+^/KDR^+^ PBMCs are earlier endothelial progenitors [34]. The role of EPC or angiogenic cells in obesity, and particularly in the Class III obese is largely unknown albeit there have been some studies which demonstrate low circulating levels of EPC in obesity with suggestions of the potential to predict CVD prevalence in this population [35], although contrary reports have also been observed [36]. In the current study, circulating levels of angiogenic cells were unchanged. This result might relate to the difference of angiogenic cells carrying different surface markers. We suggest, however, that the difference, at least partly, is the outcome of the contradicting processes occurring with inflammation suppressing the generation and mobilization of bone marrow-derived EPC [37] and highly activated adipogenesis promoting increased release of EPC from adipose tissue [38]. The latter is supported with the finding that SDF-1, secreted by adipose stromal cells [39], was significantly increased in the obese cohort. Intriguingly, the functional capacity of angiogenic cells measured as CFU-Hill colonies was significantly increased in the Class III obese group. The finding of a recent report by Hirschi *et al.* might explain this dichotomy. It reported that CFU-Hill colonies comprise primarily of monocytes and macrophages [40], and indeed what we are observing in the current study with increased CFU-Hill may simply reflect the activated inflammatory status in Class III obesity, in line with hsCRP. Our result of no correlation between cIMT or distensibility with angiogenic cells indicates that these cells may not be a cellular biomarker of CVD in Class III obesity.

It is a limitation of this study that only BMI or waist:hip ratio, but not direct measurement of body fat or body composition, was measured to reflect adiposity. Also neither insulin levels nor insulin resistance were evaluated. Further, this is a cross-sectional study, from which no casual relationship can be further explored. A future study in a cohort of subjects with BMI>40 kg/m^2^ with no overt CVD and also free of MS, *esp.* hypertension and diabetes mellitus, would be extremely useful to delineate the complexity of CVD pathophysiology in this population. Such individuals, however, are uncommon and thus difficult to identify.

In conclusion, we document in the current study that increased cIMT and reduced distensibility are present in Class III obesity. cIMT and distensibility correlate closely with traditional CVRF, adiposity and inflammatory markers, confirming the validity of these two important parameters in CVD detection in individuals with BMI>40 kg/m^2^. We also demonstrate, for the first time, elevated plasma EMP levels and unchanged circulatory CD133^+^/KDR^+^ angiogenic cells in Class III obesity.
